# Altered Default Network Resting-State Functional Connectivity in Adolescents with Internet Gaming Addiction

**DOI:** 10.1371/journal.pone.0059902

**Published:** 2013-03-26

**Authors:** Wei-na Ding, Jin-hua Sun, Ya-wen Sun, Yan Zhou, Lei Li, Jian-rong Xu, Ya-song Du

**Affiliations:** 1 Department of Radiology, Ren Ji Hospital, School of Medicine, Shanghai Jiao Tong University, Shanghai, P. R. China; 2 Department of Child & Adolescent Psychiatry, Shanghai Mental Health Center, Shanghai Jiao Tong University, Shanghai, P. R. China; Yale University, United States of America

## Abstract

**Purpose:**

Excessive use of the Internet has been linked to a variety of negative psychosocial consequences. This study used resting-state functional magnetic resonance imaging (fMRI) to investigate whether functional connectivity is altered in adolescents with Internet gaming addiction (IGA).

**Methods:**

Seventeen adolescents with IGA and 24 normal control adolescents underwent a 7.3 minute resting-state fMRI scan. Posterior cingulate cortex (PCC) connectivity was determined in all subjects by investigating synchronized low-frequency fMRI signal fluctuations using a temporal correlation method. To assess the relationship between IGA symptom severity and PCC connectivity, contrast images representing areas correlated with PCC connectivity were correlated with the scores of the 17 subjects with IGA on the Chen Internet Addiction Scale (CIAS) and Barratt Impulsiveness Scale-11 (BIS-11) and their hours of Internet use per week.

**Results:**

There were no significant differences in the distributions of the age, gender, and years of education between the two groups. The subjects with IGA showed longer Internet use per week (hours) (p<0.0001) and higher CIAS (p<0.0001) and BIS-11 (p = 0.01) scores than the controls. Compared with the control group, subjects with IGA exhibited increased functional connectivity in the bilateral cerebellum posterior lobe and middle temporal gyrus. The bilateral inferior parietal lobule and right inferior temporal gyrus exhibited decreased connectivity. Connectivity with the PCC was positively correlated with CIAS scores in the right precuneus, posterior cingulate gyrus, thalamus, caudate, nucleus accumbens, supplementary motor area, and lingual gyrus. It was negatively correlated with the right cerebellum anterior lobe and left superior parietal lobule.

**Conclusion:**

Our results suggest that adolescents with IGA exhibit different resting-state patterns of brain activity. As these alterations are partially consistent with those in patients with substance addiction, they support the hypothesis that IGA as a behavioral addiction that may share similar neurobiological abnormalities with other addictive disorders.

## Introduction

In the past decade, research has accumulated suggesting that excessive Internet use can lead to the development of a behavioral addiction [1]. Internet addiction (IA) is considered a serious threat to mental health, and excessive use of the Internet has been linked to a variety of negative psychosocial consequences. Using Young’s Diagnostic Questionnaire [YDQ][2], Sinmoes et al. found that 11% of 12-to 18-year-old adolescents in Greece fulfilled the criteria for IA [3]. Cao and Su found that 2.4% of adolescents in China were classified as having IA [4]. Shek et al. [5] reported that 19.1% of Hong Kong Chinese adolescents had IA. Accordingly, IA is prevalent across Eastern and Western societies, indicating that it is a global disorder worthy of more attention [6].

Recently, “non-substance-related behavioral addiction” has been proposed in psychiatry [7]. Contrary to the commonly held belief that addiction is specific to dependence on drugs and chemical substances, the term “addiction” has been used to refer to a range of excessive behaviors, such as gambling[8], video game playing[9], sex, and other behaviors. Although such behavioral addictions do not involve a chemical intoxicant or substance, a group of researchers have posed that some core aspects of behavioral addiction are similar to those of chemical or substance addiction[10]. Others have stated that behaviorally addicted individuals share certain symptoms with and will experience similar consequences to people addicted to alcohol and other drugs, including compulsive behaviors.

Internet addiction disorder (IAD) is a mental health problem worthy of further scientific investigation. Indeed, the prevalence of IAD has garnered so much attention that it should be included in the DSM-V[11]. Neuroimaging studies offer an advantage over traditional survey and behavioral research approaches because it makes it possible to distinguish particular brain areas that are involved in the development and maintenance of addiction. In this study, we used resting-state functional magnetic resonance imaging (fMRI) to investigate the default mode network (DMN) in adolescents with IGA. The aims of this study were 1) to investigate altered default network resting-state functional connectivity (FC), 2) to examine whether any alterations are consistent with those seen in the patients with substance addiction, and 3) to determine whether there are any relationships between altered FC and behavioral and personality measures in subjects with IAD.

## Materials and Methods

### Subjects

All subjects were recruited from the Department of Child and Adolescent Psychiatry of Shanghai Mental Health Center. They were 14 to 17 years old. We imaged seventeen subjects whose behaviors corresponded with the DSM-IV criteria for IA according to the modified Diagnostic Questionnaire for Internet Addiction (i.e., the YDQ) criteria by Beard [12]. Twenty-four age- and gender-matched healthy individuals with no personal or family history of psychiatric disorders were also imaged as the control group. All subjects were right-handed and none of them smoked.

A basic information questionnaire was used to collect demographic information such as gender, age, final year of schooling completed, and hours of Internet use per week. This study was approved by the Ethics Committee of Ren Ji Hospital of Shanghai Jiao Tong University School of Medicine. The participants and their parents or legal guardians were informed of the aims of our study before the magnetic resonance imaging (MRI) examinations were conducted. Full and written informed consent was obtained from the parents or legal guardians of each participant.

### Inclusion and exclusion criteria

All subjects underwent a simple physical examination including blood pressure and heart rate measurements, and were interviewed by a psychiatrist regarding their medical history of nervous, motor, digestive, respiratory, circulation, endocrine, urinary, and reproductive problems. They were then screened for psychiatric disorders with the Mini International Neuropsychiatric Interview for Children and Adolescents (MINI-KID)[13]. The exclusion criteria included a history of substance abuse or dependence, previous hospitalization for psychiatric disorders, or a history of major psychiatric disorders, such as schizophrenia, depression, anxiety disorder, and psychotic episodes. The subjects with IAD were not treated with psychotherapy or any medications.

The diagnostic questionnaire for IA was adapted from DSM-IV criteria for pathological gambling by Young [2]. The YDQ we used consisted of eight “yes” or “no” questions translated into Chinese. It included the following questions: (1) Do you feel absorbed in the Internet, as indexed by remembering previous online activity or the desire for the next online session? (2) Do you feel satisfied with your Internet use if you increase your amount of online time? (3) Have you failed to control, reduce, or quit Internet use repeatedly? (4) Do you feel nervous, temperamental, depressed, or sensitive when trying to reduce or quit Internet use? (5) Do you stay online longer than originally intended? (6) Have you taken the risk of losing a significant relationship, job, educational, or career opportunity because of the Internet? (7) Have you lied to your family members, therapist, or others to hide the truth of your involvement with the Internet? (8) Do you use the Internet as a way of escaping from problems or of relieving an anxious mood (e.g., feelings of helplessness, guilt, anxiety, or depression)? Young asserted that five or more “yes” responses to the eight questions indicate a dependent user. Later, Beard and Wolf [12] modified the YDQ criteria to state that respondents who answered “yes” to questions 1 through 5 and at least one of the remaining three questions should be classified as suffering from IA.

### Behavioral and personality assessments

Four questionnaires were used to assess the participants’ behavioral and personality features, namely the Chen Internet Addiction Scale (CIAS)[14], Self-Rating Anxiety Scale (SAS)[15], Self-rating Depression Scale (SDS) [16], and Barratt Impulsiveness Scale-11 (BIS-11) [17]. All questionnaires were initially constructed in English and then translated into Chinese.

### MRI acquisition

MRI was conducted using a 3T MRI scanner (GE Signa HDxt 3T, USA). A standard head coil with foam padding was used to restrict head motion. During resting-state fMRI, the subjects were instructed to keep their eyes closed, remain motionless, stay awake, and not to think of anything in particular. A gradient-echo echo-planar sequence was used for functional imaging. Thirty-four transverse slices [repetition time (TR) = 2000 ms, echo time(TE) = 30 ms, field of view (FOV) = 230×230 mm, 3.6×3.6×4 mm voxel size] aligned along the anterior commissure-posterior commissure line were acquired. Each fMRI scan lasted 440 s. Several other sequences were also acquired, including (1) a sagittal T1-weighted 3D-magnetization prepared rapid acquisition gradient echo sequence [TR = 9.4 ms, TE = 4.6 ms, flip angle = 15°, FOV = 256×256 mm, 155 slices,1×1×1 mm voxel size], (2) axial T1-weighted fast field echo sequences [TR = 331 ms, TE = 4.6 ms, FOV = 256×256 mm, 34 slices, 0.5×0.5×4 mm voxel size], and (3) axial T2W turbo spin-echo sequences [TR = 3013 ms, TE = 80 ms, FOV = 256×256 mm, 34 slices, 0.5×0.5×4 mm voxel size].

### Image analysis

Two-sample *t*-tests were used for group comparisons to examine demographic differences between the two groups, and χ^2^-tests were used for gender comparisons. A two-tailed *p*-value of 0.05 was considered statistically significant for all analyses.

Structural brain MRI scans (T1- and T2-weighted images) were inspected by two experienced neuroradiologists. No gross abnormalities were observed in either group. Functional MRI preprocessing was performed using the Data Processing Assistant for Resting-State fMRI V 2.0 (YAN Chao-Gan, http://www.restfmri.net), which is integrated with MRIcroN toolset (Chris Rorden, http://www.mricro.com), statistical parametric mapping (SPM5; Wellcome Department of Imaging Neuroscience, London, UK), and the Resting-State fMRI Data Analysis Toolkit (REST V1.8 software, Song et al., http://www.restfmri.net).

The first 10 volumes of each functional time-series were discarded because of the instability of the initial MRI signal and the initial adaptation of participants to the situation. Data from each fMRI scan contained 220 time points, and the remaining 210 images were preprocessed. The images were subsequently corrected for slice timing and realigned to the first image by rigid-body head movement correction (patient data exhibiting movement greater than 1 mm with maximum translation in *x*, *y*, or *z*, or 1° maximum rotation about the three axes were discarded). No participant was excluded because of movement. The functional images were normalized into standard stereotaxic anatomical Montreal Neurological Institute (MNI) space. The normalized volumes were resampled to a voxel size of 3 mm×3 mm×3 mm. The echo-planar images were spatially smoothed using an isotropic Gaussian filter of 4 mm full width at half maximum.

The time-series in each voxel was detrended to correct for linear drift over time. Nine nuisance covariates (time-series predictors for global signal, white matter, cerebrospinal fluid, and the six movement parameters) were sequentially regressed from the time-series[18], [19]. Subsequently, temporal filtering (0.01–0.08 Hz) was applied to the time-series of each voxel to reduce the impact of low-frequency drifts and high-frequency noise[8], [20]–[22]

The PCC template, which consisted of Brodmann’s areas 29, 30, 23, and 31, was selected as the region of interest (ROI) using WFU-Pick Atlas software[23]. The blood oxygenation level-dependent signal time-series in the voxels within the seed region were averaged to generate the reference time-series. For each subject and seed region, a correlation map was produced by computing the correlation coefficients between the reference time-series and the time-series from all other brain voxels. Correlation coefficients were then converted to *z* values using Fisher’s *z*-transform to improve the normality of the distribution[22]. The individual *z*-scores were entered into SPM5 for a one-sample *t*-test to determine the brain regions with significant connectivity to the PCC within each group. Individual scores were also entered into SPM5 for random effect analysis and two-sample *t*-tests to identify the regions exhibiting significant differences in connectivity to the PCC between the two groups. Multiple comparison correction was performed using the AlphaSim program in the Analysis of Functional Neuroimages software package, as determined by Monte Carlo simulations. Statistical maps of the two-sample *t*-test were created using a combined threshold of *p*<0.05 and a minimum cluster size of 54 voxels, yielding a corrected threshold of *p*<0.05. Regions exhibiting statistically significant differences were masked on MNI brain templates. The CIAS developed by Chen contains 26 items on a 4-point Likert scale. Its total score ranges from 26 to 104 and represents the severity of Internet addiction. Previous studies have shown that patients with IA have impaired impulse control [24]. Therefore, contrast images representing areas of correlation between activity in the seed region and CIAS and BIS-11 scores and hours of Internet use per week (hours) were generated for the 17 subjects with IGA to assess the relationships between the severity of IGA symptoms, impulsivity, and PCC connectivity, using a threshold of *p*<0.05 AlphaSim corrected.

## Results

### Demographic and behavioral measures

Table 1 lists the demographic and behavioral measures for the IGA and control subjects. There were no significant differences in the distributions of age, gender, and years of education between the two groups. The subjects with IGA engaged in more hours of Internet use per week (p<0.0001) and had higher CIAS (p<0.0001) and BIS-11 (p = 0.01) scores than the controls. No differences in SAS or SDS scores were found between the groups.

**Table 1:** Demographic and behavioral characteristics of the included participants.

	Adolescent internet addiction disorder group (n = 17)	Control group (n = 24)	p value
	(Mean ± SD)	(Mean ± SD)	
Age(yeas)	16.94±2.73	15.87±2.69	0.22
Gender (M/F)	13/4	16/8	0.46
Education (yeas)	9±2.67	8.96±2.84	0.96
Time for internet use per week (hours)	26.44±21.47	10.50±11.60	<0.0001
Chen Internet Addiction Scale (CIAS)	64.59±6.43	45.70±7.81	<0.0001
Self-Rating Anxiety Scale (SAS)	45.12±7.41	42.30±5.34	0.15
Self-rating depression scale (SDS)	50.76±7.93	47.13±7.31	0.16
Barratt Impulsiveness Scale-11 (BIS-11)	62.53±7.12	56.25±7.07	0.01

Abbreviation. SD: standard deviation.

Two-sample t test was used for group comparisons but chi-square was used for gender comparison.

### Between-group analysis of PCC connectivity

**Figure 1:**
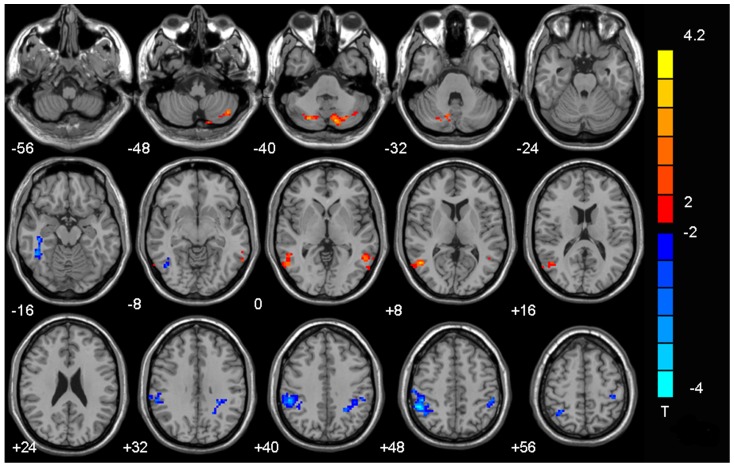
Significant between-group differences in functional connectivity between healthy control subjects and those with IGA. Compared with the control group, the subjects with IGA exhibited increased FC in the bilateral cerebellum posterior lobe and middle temporal gyrus. Several regions also exhibited decreased connectivity, including the bilateral inferior parietal lobule and right inferior temporal gyrus. (*p*<0.05, AlphaSim-corrected). The t-score bars are shown on the right. Red indicates IGA>controls and blue indicates IAD<controls. Note: The left part of the figure represents the patient’s right side. IGA =  Internet gaming addiction; FC  =  functional connectivity.

A between-group analysis was performed using a two-sample t-test in SPM5. Compared with the control group, subjects with IGA exhibited increased FC in the bilateral cerebellum posterior lobe and middle temporal gyrus. Their bilateral inferior parietal lobule and right inferior temporal gyrus exhibited decreased connectivity (Table 2 and Figure 1).

**Table 2:** Significant between-group differences in functional connectivity between specific brain regions and the posterior cingulate cortex.

	Peak MNI coordinate region	Peak MNI coordinates			Number of cluster voxels	Peak *T* value
		x	y	z		
1	Left cerebellum posterior lobe	−12	−78	−39	89	3.52
2	Right cerebellum posterior lobe	24	−75	−36	55	4.03
3	Left middle temporal gyrus	−54	−54	0	71	3.05
4	Right middle temporal gyrus	51	−60	9	111	3.52
5	Right inferior temporal gyrus	45	−45	−15	54	−3.26
6	Right inferior parietal lobule	57	−27	51	324	−4.07
7	Left inferior parietal lobule	−36	−39	36	135	−3.63

(p<0.05, AlphaSim-corrected, extent threshold = 54 voxel)

Note: T>0 indicated IGA>controls in functional connectivity in PCC

T<0 indicated IGA<controls in functional connectivity in PCC

IGA = internet gaming addiction

### Correlation between PCC connectivity and CIAS and BIS-11 scores and hours of Internet use per week in subjects with IGA

**Figure 2:**
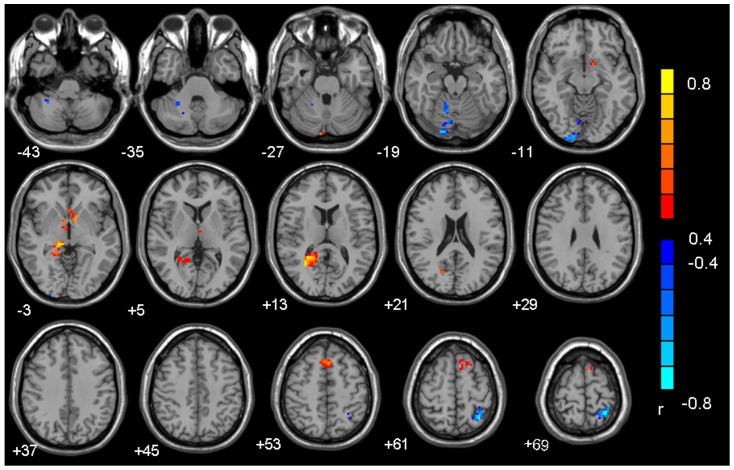
Brain regions in which functional connectivity with the PCC correlated with CIAS scores significantly in the subjects with IGA. (*p*<0.05, AlphaSim-corrected).

Connectivity with the PCC was positively correlated with CIAS scores in the right precuneus, posterior cingulate gyrus, thalamus, caudate, nucleus accumbens, supplementary motor area (SMA), and lingual gyrus, and it was negatively correlated in the right cerebellum anterior lobe and left superior parietal lobule (Table 3 and Figure 2). There was no significant correlation between connectivity with the PCC and BIS-11 scores or hours of Internet use per week.

**Table 3:** Brain regions in which functional connectivity with the PCC correlated with CIAS scores in the subjects with IGA.

	Peak MNI coordinate region	Peak MNI coordinates			Number of cluster voxels
		x	y	z	
1	Right cerebellum anterior lobe	27	−51	−33	98
2	Right lingual gyrus	9	−93	−30	99
3	Right precuneus/posterior cingulate	30	−57	12	219
4	Right thalamus/caudate/nucleus accumbens	9	0	0	95
5	Right supplementary motor area(SMA)	3	21	57	80
6	Left superior parietal lobule	−30	−54	63	95

(p<0.05, AlphaSim-corrected)

Note: PCC  =  posterior cingulate cortex; IGA  = internet gaming addiction; CIAS  =  Chen Internet Addiction Scale

Note: The right part of the figure represents the patient’s left side. PCC = posterior cingulate cortex; IGA =  Internet gaming addiction; CIAS =  Chen Internet Addiction Scale.

## Discussion

Accumulating research suggests that excessive Internet use can lead to the development of a behavioral addiction [25], [26]. People experiencing IAD show clinical features that include craving, withdrawal, and tolerance[11], [27], increased impulsiveness [28], and impaired cognitive performance in tasks involving risky decision-making[29]. Some of these symptoms have been traditionally associated with substance-related addictions [30]. IA comprises a heterogeneous spectrum of Internet activities that can result in illness, such as gaming, shopping, gambling, or social networking. Gaming represents a part of the postulated construct of IA, and gaming addiction appears to be the most widely studied form of IA to date [31]. In recent years, IAD has become more prevalent worldwide and the recognition of its devastating impact on users and society has rapidly increased. However, the neurobiological mechanism of IAD has not been fully elucidated.

Some researchers support the claim that IAD shares similar neurobiological abnormalities with other addictive disorders. Hou et al.,[32] found dopamine transporter (DAT) expression levels in the striatum were significantly lower in individuals with IAD using 99mTc-TRODAT-1 single photon emission computed tomography brain scans. DATs play a critical role in the regulation of striatal synaptic dopamine levels [33], and have been used as markers of the dopamine terminals [34]. A reduced number of cell membrane DATs may possibly reflect pronounced striatal dopamine terminal loss or brain dopaminergic system impairments, which has also been found in substance-related addiction [35]. Because increased extracellular dopamine in the striatum is associated with subjective descriptions of reward, such as high and euphoria [36], individuals with IAD may also experience euphoria as extracellular dopamine levels in the striatum increase. Patients with pathological gambling demonstrated a high level of dopamine in the ventral striatum during gambling[37]. Positron emission tomography imaging studies have found increased release of dopamine in the striatum during video game playing [38].

Some researchers [39]–[44] have applied resting-state fMRI in patients with substance dependence to further understand its mechanisms and help explain its behavioral and neuropsychological deficits. A number of studies have identified key brain regions thought to participate in addiction disorders, such as the nucleus accumbens [45], dorsal striatum, and prefrontal cortex (PFC) [46], [47]. The results provided by Zhang et al.,[39] showed activation pattern differences between heroin-dependent and healthy subjects, in regions including the orbitofrontal cortex (OFC), cingulate gyrus, frontal and para-limbic regions such as the anterior cingulate cortex (ACC), hippocampal/parahippocampal regions, amygdala, caudate, putamen, posterior insula, and thalamus. These regions are involved in brain networks underpinning reward, motivation, learning and memory, and the control of other circuits. Tanabe et al.,[40]found that nicotine consumption was associated with decreased activity in regions within the DMN and increased activity in extra-striate regions. They suggested that these effects of nicotine, in the absence of visual stimuli or effortful processing, suggest that its cognitive effects may involve a shift from networks that process internal information to those that process external information. Another study reported that smokers had greater coupling versus non-smokers between left fronto-parietal and medial prefrontal cortex (mPFC) networks. Smokers with the greatest mPFC-left fronto-parietal coupling had the most dorsal striatum smoking cue reactivity as measured during an fMRI smoking cue reactivity paradigm[41]. A study performed by Ko CH et al., [48] evaluated brain correlates of cue-induced craving to play online games in subjects with IGA. Their results showed that the bilateral dorsolateral prefrontal cortex (DLPFC), precuneus, left parahippocampus, posterior cingulate and right anterior cingulate were activated in response to gaming cues in the IGA group in a manner that was stronger than in the control group. Thus, these findings suggest that the neurobiological underpinnings of IGA are similar to those of substance use disorders.

Based on the model proposed by Volkowet al.,[49] a number of neurobiological systems may mediate cue-induced gaming craving. These include visual processing areas such as the occipital lobe or precuneus that link gaming cues to internal information, and memory systems that include the hippocampus, parahippocampus, or amygdala and that provide emotional memories and contextual information for the gaming cues. They also include reward systems such as the limbic system and posterior cingulate that allow for the evaluation of gaming-related information and provide expectations and reward significance, and they include motivation systems such as the anterior cingulate and orbital frontal lobe that control the desire for gaming. Finally, these systems include executive systems such as the DLPFC and prefrontal cortex that allow one to form a plan to get online for gaming.

We found subjects with IGA exhibited increased FC in the bilateral cerebellum posterior lobe and middle temporal gyrus. The bilateral inferior parietal lobule and right inferior temporal gyrus exhibited decreased connectivity compared with the control group. Connectivity with the PCC was positively correlated with CIAS scores, which are related to the severity of the IGA, in the right precuneus, posterior cingulate gyrus, thalamus, caudate, nucleus accumbens, supplementary motor area, and lingual gyrus. They were negatively correlated with the right cerebellum anterior lobe and left superior parietal lobule.

The functions of the cerebellum are not limited to movement and balance, as it also plays an important role in emotional and cognitive processes [50], [51]. It receives input from sensory systems and other parts of the brain, and integrates these inputs to fine-tune motor activity[52]. The posterior cerebellum is predominantly involved in cognitive regulation[53], signal processing, and storage of relevant auditory-verbal memory processes[54]. Blood flow (rCBF) apparently increases in the cerebellum when craving is induced by a cocaine cue [55]. Paradiso and Takeuchi contended that cerebellar activation may be related to emotional processes and attention during cue-induction [56], [57]. In research regarding alterations in regional homogeneity (Reho) of resting-state brain activity in subjects with IGA[58], there was increased Reho in the left posterior cerebellum. This suggests that the cerebellum plays an important role in craving induced by IGA, especially during preparation, execution, working memory[59], and fine-motor processes modulated by extrapyramidal systems. We found increased FC in the bilateral posterior cerebellum, but a negative correlation in the right cerebellum anterior lobe with CIAS scores. Although the locations were different, in terms of functions of the cerebellum, there was a more important distinction along the medial-to-lateral dimension. As such, this contention cannot be confirmed in this present study and needs to be investigated by a follow-up study.

The bilateral middle temporal gyrus showed increased FC in the subjects with IGA, but the right inferior temporal gyrus showed decreased FC. The inferior temporal gyrus is one of the higher levels of the ventral stream of audio and visual processing, and is associated with the representation of complex object features[60]. Dong et al. found decreased Reho in the inferior temporal gyrus, and they wrote that decreased ReHo in visual- and auditory-related brain regions may suggest that the decreased synchronization in subjects with IGA may be the result of a long duration of game playing [58]. Our results are partially consistent with this hypothesis, which should be investigated in future studies.

We found decreased FC in the bilateral inferior parietal lobule, and the FC of the left superior parietal lobule including the PCC was negatively correlated with CIAS scores. Various studies have found that the parietal lobe has a concerted involvement in visuospatial tasks. Position changes of the watched object can lead to strong bilateral activation of the superior parietal cortex[61]. Olson et al.,[62]discovered that the parietal lobe played a dominant role in short-term memory. Furthermore, some researchers have hypothesized that the parietal cortex may play a role in regulating attention or withholding motor responses during response inhibition tasks[63], [64].

Connectivity with the PCC was positively correlated with CIAS scores in the right precuneus, posterior cingulate gyrus, thalamus, caudate, nucleus accumbens, SMA, and lingual gyrus. Most of these regions are part of the reward system[65]. The precuneus is associated with visual imagery, attention, and memory retrieval. It participates in the visual process and integrates related memories. Research suggests that the precuneus is activated by gaming cues, integrates retrieved memories, and contributes to cue-induced craving for online gaming[66]. As a central component of the proposed DMN, the PCC is implicated in attentional processes. Previous studies have demonstrated that PCC neurons respond to reward receipt, magnitude, and visual-spatial orientation [67], [68]. Previous studies have found that the thalamus plays an important role in reward processing [69] and goal-directed behaviors, along with many other cognitive and motor functions [70]. Dong et al.,[71] found abnormal thalamo-cortical circuitry in subjects with IGA, suggesting implications for reward sensitivity. Activation of the striatum has been reported during reward prediction, tracking reward prediction errors, and in more complex gambling paradigms [72], [73] Recently, it has been proposed that the striatum is involved in coding stimulus saliency rather than having an exclusive role in reward processing per se[74]. Action preparation for reward could modulate activity in brain regions such as the dorsal striatum.[75]–[77]. Studies of response inhibition using fMRI have consistently found that the pre-SMA is critical for the selection of appropriate behaviors, including executing appropriate and inhibiting inappropriate responses [78].

The lingual gyrus is a visual area. We previously found differences in grey matter density in the lingual gyrus in healthy subjects as compared to those with IAD [79], [80]. This visual associative area has been implicated in schizophrenia[80]–[83]. One study[83] demonstrated increased gyrification and reduced cortical thickness of the lingual gyrus, which extended previous findings of aberrant morphology of the lingual region in schizophrenia[84]. The right parahippocampus and lingual gyrus has been shown to be involved in right hemispheric dominated networks mediating emotional functions [85]. In addition, Seiferth et al. [86] showed that the right lingual gyrus was hyperactivated during emotion discrimination in high-risk subjects.

Abnormalities in the FC of the PCC with the mPFC and ACC were not found in the present study. This may be partly attributable to the limited sample size and the mild severity of IAD in the participants as compared to subjects we examined previously [25], [48], [57].

### Limitations of the study

There are several limitations that should be mentioned in this study. First, the diagnosis of IAD was mainly based on results of self-reported questionnaires, which could cause some error classification. Second, the sample size was relatively small, which could reduce the power of the statistical analyses and hamper generalization of the findings. Owing to this limitation, the reported results should be considered preliminary, and they should be replicated in future studies with larger sample sizes. Third, as a cross-sectional study, our results do not clearly demonstrate whether the psychological features preceded the development of IAD or were a consequence of the overuse of the Internet. Therefore, future prospective studies should clarify the causal relations between IAD and psychological measures. Last, to elucidate the shared neurobiology of substance addiction and behavioral addictions such as IGA, further research investigating patients from both clinical populations should be conducted.

## Conclusions

This paper describes a preliminary study of FC in adolescents with IGA. Our results suggested adolescents with IGA exhibited different resting-state patterns of neuronal activity. The alterations were partially consistent with those that have been reported in patients with substance addiction. Therefore, these results support the hypothesis that IGA as a behavioral addiction may share similar neurobiological abnormalities with other addictive disorders.
